# Construction of a versatile in vitro cultivation screening platform using human oral microbiota

**DOI:** 10.1111/1758-2229.13243

**Published:** 2024-02-29

**Authors:** Kengo Sasaki, Yasunobu Takeshima, Ayami Fujino, Junya Yamashita, Akira Kimoto, Daisuke Sasaki, Akihiko Kondo, Masaya Akashi, Ryo Okumura

**Affiliations:** ^1^ Bio Palette Co., Ltd Kobe Japan; ^2^ Department of Oral and Maxillofacial Surgery Kobe University Graduate School of Medicine Kobe Hyogo Japan; ^3^ Graduate School of Science, Technology and Innovation, Kobe University Kobe Hyogo Japan

## Abstract

We developed a simulation model of human oral microbiota using Bio Palette oral medium (BPOM) containing 0.02% glucose and lower bacterial nitrogen sources, derived from saliva and dental plaque. By decreasing the concentration of Gifu anaerobic medium (GAM) from 30 to 10 g L^−1^, we observed increased ratios of target pathogenic genera, *Porphyromonas* and *Fusobacterium* from 0.5% and 1.7% to 1.2% and 3.5%, respectively, in the biofilm on hydroxyapatite (HA) discs. BPOM exhibited the higher ratios of *Porphyromonas* and *Fusobacterium*, and amplicon sequence variant number on HA, compared with GAM, modified GAM and basal medium mucin. Mixing glycerol stocks of BPOM culture solutions from four human subjects resulted in comparable ratios of these bacteria to the original saliva. In this simulation model, sitafloxacin showed higher inhibitory effects on *P. gingivalis* than minocycline hydrochloride at a low dosage of 0.1 μg mL^−1^. Probiotics such as *Streptococcus salivarius* and *Limosilactobacillus fermentum* also showed significant decreases in *Porphyromonas* and *Fusobacterium* ratios on HA, respectively. Overall, the study suggests that BPOM with low carbon and nutrients could be a versatile platform for assessing the efficacy of antibiotics and live biotherapeutics in treating oral diseases caused by *Porphyromonas* and *Fusobacterium*.

## Graphical abstract

This study aims to develop an in vitro simulation model of human oral microbiota that includes target pathogens and maintains microbial diversity from saliva and dental plaque. To achieve this aim, a new oral medium called Bio Palette oral medium was created with low levels of sugar and nitrogen sources. This resulting model is versatile and can efficiently screen antibiotics and live biotherapeutics.

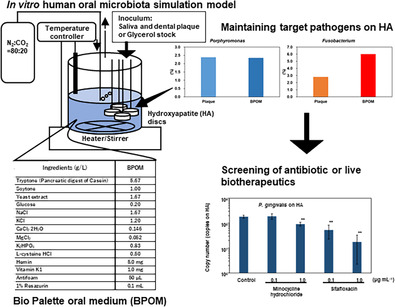

## INTRODUCTION

Oral microbiota, consisting of microorganisms in the human oral cavity, is the second largest microbial community in humans, following the gut (Verma et al., 2018). Microorganisms form complex matured multispecies biofilm communities, particularly on tooth surfaces, as the oral cavity comprises various surfaces (Lamont et al., 2018). Dysbiotic oral microbial communities are strongly associated with oral diseases such as periodontitis and dental caries (Lamont et al., 2018). Recent studies suggest that dysbiosis of the oral microbiota also plays a role in the onset and progression of major human diseases such as cardiovascular and neurodegenerative diseases (Giordano‐Kelhoffer et al., 2022).

In the diseased state, pathogenic dental plaque biofilm accumulating on the tooth surface destroys supporting tissues through unresolved inflammation (Lasserre et al., 2018). The first step in dental plaque formation is the attachment of a pellicle, a thin film derived from salivary glycoproteins, to a clean tooth surface (Huang et al., 2011). The initial dental plaque consists mainly of early colonizers, such as Gram‐positive aerobes, including *Streptococcus* spp. and *Actinomyces* spp. (Lasserre et al., 2018), which provide specific binding sites for secondary colonizers, including *Fusobacterium nucleatum*, and periopathogens, including *Porphyromonas gingivalis* (Huang et al., 2011). *F. nucleatum* is a crucial bridging bacterium that facilitates the integration of periopathogens into oral biofilms (Park et al., 2016).

Over the years, in vitro and oral biofilm models have improved, expanding our knowledge of the complex biofilm structure and its pathogenic mechanisms and testing antimicrobial and anti‐biofilm strategies (Brown et al., 2019; Sánchez et al., 2021). Two types of inoculums have been collected to generate laboratory biofilm dental plaque models, including biological samples, such as saliva or dental plaque, and defined mixed bacterial species (Sissons, 1997). In addition, two types of culturing methods have been developed: continuous cultures under controlled conditions and batch process (Brown et al., 2019; Mira et al., 2019; Salli & Ouwehand, 2015). Batch models contain surfaces for oral biofilm growth, such as multi‐well plates or discs (Brown et al., 2019; Salli & Ouwehand, 2015).

This study employed the batch method for an in vitro human oral microbiota simulation model, as it requires small amounts of reagents (Salli & Ouwehand, 2015). Saliva and plaque samples were used as inoculums, and exogenous *P. gingivalis* was added if necessary, as its abundance is lower in periodontally healthy subjects than in periodontitis patients (Amano et al., 2000). Hydroxyapatite (HA) discs were introduced into the systems to assess biofilm formation (Sudo, 1977). A synthetic saliva medium was developed to mimic the artificial dental plaque biofilm as closely as possible to natural human biofilm and to test the behaviour of antimicrobial agents, particularly on potential oral pathogens (Sim et al., 2016; Tian et al., 2010). The present study aimed to develop an optimal synthetic medium, Bio Palette oral medium (BPOM), which can be used in batch culture. We propose a new concept for synthetic medium for a batch culture that requires fewer nutrients to hold target oral pathogens closer to natural human dental plaque and saliva. To validate the usefulness of this new synthetic medium, BPOM, microbiota on HA discs and in culture solution were analysed using next‐generation sequencing technologies targeting the 16S rRNA gene. In addition, to improve reproducibility, glycerol stock mixtures were prepared as inoculum from different human subjects rather than preparing human inoculum each time, which can be laborious and results in differing outcomes person by person.

## EXPERIMENTAL PROCEDURES

### Collection of samples

Nine participants, including three patients diagnosed with periodontal disease and six volunteers, were recruited to undergo bleeding on probing and probing pocket depth measurement at the Kobe University Hospital. Participants with probing pocket depth measuring ≥5 mm were defined as patients with periodontal disease, whereas the remaining participants were defined as volunteers. All samples were collected under resting conditions at least 1 h after eating. Saliva was collected from each participant for 5 min using a cup, and 5 mL of the collected saliva was diluted 1:1 with PBS. In addition, dental plaque (including gingival crevicular fluid) was collected from the deepest periodontal pocket using a paper point and from the surface of the cervical area using a sterile probe. These paper points and probes were submerged in 500 μL of PBS, and both saliva and dental plaque samples were used within 24 h. Written informed consent was obtained from all participants before specimen collection. The sampling procedure was approved by the Bio Palette Co., Ltd. Ethics Committee with references BE‐2021‐01 (for sampling from humans), BE‐2021‐02 (for model construction) and BE2021‐03 (for reproduction of B200301) and by Kobe University with reference B200301.

A portion of the collected saliva was sterilized by ultraviolet radiation for 1 h, and the sterilized saliva samples were stored at −20°C until use in pellicle formation.

### Construction of in vitro human oral microbiota simulation model

**FIGURE 1:**
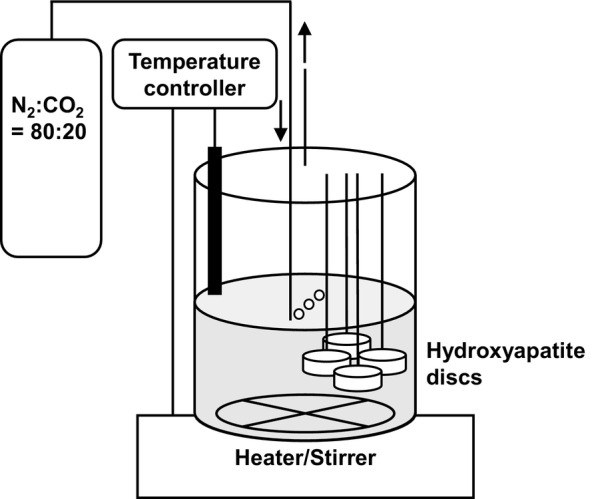
Visual overview of an in vitro human oral microbiota simulation model. Each vessel set up hydroxyapatite discs in it and contained an agitated autoclaved medium, kept at 37°C and purged with a gas mixture.

This study utilized a Bio Jr.8 multichannel fermenter (ABLE, Tokyo, Japan) consisting of eight parallel and independent vessels (Figure 1) (Sasaki et al., 2019). Four HA discs (APP‐610, Hoya Technological Co., Tokyo, Japan) were immersed in sterile human saliva for 2 h at 37°C under aerobic conditions and then affixed to each vessel's stainless‐steel wire. The medium (pH: approximately 6.0) (Table S1, Supporting Information) was added to each vessel, which contained the following components per litre of BPOM: 5.67 g of Bacto‐Tryptone (Becton, Dickinson, and Company, Sparks, MD, USA), 1.00 g of Bacto‐Soytone (Becton, Dickinson), 1.67 g of Bacto‐Yeast Extract (Becton, Dickinson), 0.20 g of glucose, 1.67 g of NaCl, 0.83 g of K_2_HPO_4_, 0.50 g of L‐cysteine hydrochloride, 1.0 mL of Hemin stock solution (American Type Culture Collection [ATCC] medium: 2722), 0.2 mL of Vitamin K1 stock (ATCC medium: 2722), 50 μL of antiform PE‐M (FUJIFILM Wako Pure Chemical, Osaka, Japan) and 0.1 mL of 1% resazurin–sodium salt solution. After autoclaving at 115°C for 15 min, 1.20 g L^−1^ of KCl, 0.15 g L^−1^ of CaCl_2_·H_2_O and 0.05 g L^−1^ of MgCl_2_·H_2_O were added after filter sterilization. Gifu anaerobic medium (GAM)‐based medium included GAM (Code 05422, Nissui Pharmaceutical Co., Ltd., Tokyo, Japan), and modified GAM (MGAM)‐based medium included MGAM (Code 05433, Nissui). For the preparation of basal medium mucin (BMM)‐based medium, Bacto Protease Peptone (Becton, Dickinson) and type II porcine stomach mucin (Sigma Aldrich, St Louis, MO, USA) were used.

The medium was prepared by purging it with a filter‐sterilized gas mixture of N_2_ and CO_2_ (80:20) at 37°C for 1 h, with a flow rate of 10 mL min^−1^ and 100 rpm, prior to cultivation. In addition, the medium was inoculated with 400 μL of saliva and 50 μL of dental plaque suspension. During the 72 h cultivation process, the medium was continuously agitated at 100 rpm and purged with a filter‐sterilized gas mixture.

### Sampling and DNA extraction

After 72 h of cultivation in vitro, culture solutions were sampled. To collect the biofilm, HA discs were sonicated at 38 kHz for 30 s in 1 mL PBS using an ultrasonic wave apparatus (SND US‐109). DNA extraction from human inoculum (saliva and plaque), culture solutions and biofilms on the HA disc followed the protocol of Matsuki et al. (2002) The resulting DNA pellets were dissolved in Tris‐EDTA buffer (10 mM Tris–HCl, 1.0 mM Ethylenediamine tetraacetic acid) and stored at −20°C until analysis.

### 16S rRNA gene amplification and sequencing

Polymerase chain reaction (PCR) and amplicon pool preparation were performed following the 16S rRNA gene Metagenomic Sequencing Library Preparation Illumina protocol (Part # 15044223 Rev. B) with bacteria‐specific primers targeting the 16S rRNA gene V3 and V4 regions (Klindworth et al., 2013). DNA was sequenced on a MiSeq Sequencer using the 2 × 300 bp paired‐end protocol according to the manufacturer's instruction (Illumina, San Diego, CA, USA). The sequences were trimmed using the FASTX‐Toolkit (ver. 0.0.14) (Hannon, 2014) and filtered using Sickle version 1.33 with quality and length thresholds of 20 and 130, respectively (Joshi & Fass, 2011). The DADA2 plug‐in for QIIME 2 (v. 2021.8) was utilized to perform quality filtering and chimaera removal and to create a feature table of read abundance per amplicon sequence variant (ASV) by sample (Callahan et al., 2016). Taxonomic was assigned to ASVs using the naive Bayes classifier plug‐in (Bokulich et al., 2018) and by importing Greengenes 16S rRNA Database (release 13.8) to QIIME 2.

### Real‐time PCR analysis

Real‐time PCR was performed using Light Cycler 96 System (Roche, Switzerland) by amplifying with a probe and primer sets that targeted *P. gingivalis*, as described previously (Martin et al., 2002).

### Preparation of glycerol stock

Saliva and dental plaque were collected from one patient and three volunteers (V1, V2, V3 and P1) (Figure S1, Supporting Information). To prepare the first glycerol stocks (V1‐1, V2‐1, V3‐1 and P1‐1), glycerol solutions (final 16% [v/v]) were added to each BPOM, which was inoculated with each human inoculum, cultured for 72 h and stored at −80°C until use. Second glycerol stocks of each culture solution (V1‐2, V2‐2, V3‐2 and P1‐2) were prepared by inoculating 100 μL of each first glycerol stock into a BPOM. In addition, duplicate glycerol stock of culture solution (Mix1‐1 and Mix2‐1) was prepared by inoculating 100 μL of the first glycerol stock mixture containing four subjects in equal amounts into the BPOM. Finally, the duplicate glycerol stock of the culture solution (Mix1‐2 and Mix2‐2) was prepared by inoculating 100 μL of each glycerol stock prepared from the mixture into the BPOM.

### Antibiotics administration

*P. gingivalis* ATCC 33277 was anaerobically precultured at 37°C in ATCC medium 2722. The model culture system with BPOM was initiated using 100 μL of glycerol stock, Mix1‐2 and 10^7^ cells mL^−1^ of *P. gingivalis*.

Minocycline hydrochloride (Tokyo Chemical Industry Co., Tokyo, Japan) and sitafloxacin (Toronto Research Chemicals Inc., Toronto, Ontario, Canada) were dissolved in N, N‐dimethylformamide and 0.1 N HCl, respectively. After 6 h of cultivation, 0.1 or 1.0 μg mL^−1^ of minocycline hydrochloride or sitafloxacin was added to the in vitro model, and no administration was used as the control. Cultivation lasted for 72 h.

### Probiotics administration

To isolate *Streptococcus salivarius*, human saliva was plated onto an autoclaved reinforced clostridial medium (CM0149, Oxoid, Basingstoke, Hants, UK) with 1% agar (Nacalai Tesque, Kyoto, Japan) and incubated at 37°C under aerobic conditions for 1 day. In addition, a single colony was subcultured in brain heart infusion (BHI) medium (BD Co., USA) and stored as a stock culture at −80°C with 16% (vol vol^−1^) glycerol.

For isolation of *Limosilactobacillus fermentum*, a human faecal sample was cultured in a medium containing 20 g L^−1^ of young barley leaf extract (Genryoya, Osaka, Japan) and 0.5 g L^−1^ of L‐cysteine hydrochloride and cultured at 37°C under anaerobic condition (N_2_: 80%, H_2_: 10%, CO_2_: 10%) for 7 days. This culture solution was mixed with the same medium, containing 1.5 g L^−1^ of agar. A single colony was picked from this pour plate, subcultured in reinforced clostridial medium and stored as a stock culture at −80°C with glycerol (16% [v/v]).

*S. salivarius* and *L. fermentum* were precultured overnight in BHI and Difco Lactobacilli MRS medium (Becton, Dickinson), respectively. At 0 h of cultivation, 4 × 10^4^ cells mL^−1^ of *S. salivarius* or *L. fermentum* was administrated into the in vitro model. Control was set without any administration. Cultivation was conducted for 72 h.

### Statistical analysis

The data were compared between two groups using Student's *t*‐test or paired *t*‐test, with R v. 4.2.1 (The R Foundation, Vienna, Australia) being utilized for the analysis.

## RESULTS

### Effect of medium concentration on target microorganisms in an in vitro simulation model of human oral microbiota

Our in vitro oral microbiota simulation model aimed to replicate the formation of dental plaque biofilm (Rosan & Lamont, 2000) on HA discs. The HA discs were first immersed in the sterile human saliva to form pellicles before being placed in the reactor system (Figure 1). Next, the culture medium was inoculated with human saliva and dental plaque obtained from a volunteer. Oral bacteria from the inoculum grew anaerobically in a planktonic state in the culture solution. Subsequently, initial colonizers adhered to the salivary pellicle on HA discs, followed by secondary colonization through interbacterial adhesion. This process led to the gradual construction of dental plaque biofilm on HA discs.

The effect of medium concentration on the microbiota composition in the in vitro oral microbiota simulation model was investigated in this study. GAM was prepared at concentrations of 10, 20 and 30 g L^−1^, and to mimic the compositions of saliva, including metal ions, sodium chloride, potassium chloride, calcium chloride hydrate, magnesium chloride and dipotassium phosphate were added to the GAM (Table S1, Supporting Information) (Kawaguchi‐Uemura et al., 2018).

The bacterial inoculums and microbiota composition in the in vitro human oral microbiota simulation model were analysed by sequencing the bacterial 16S rRNA gene at 72 h of cultivation. *P. gingivalis* and *F. nucleatum* are potential pathogens associated with periodontal disease (Andrade et al., 2019), and thus, target bacterial genera should be maintained in the model. The ratios of bacteria related to *Porphyromonas* and *Fusobacterium* genera to all bacteria on HA discs and in culture solutions were highest at 10 g L^−1^ of GAM, compared with at 20 and 30 g L^−1^ of GAM (Table 1). Similarly, the microbial diversity index and Shannon index on HA discs and in culture solutions were highest at 10 g L^−1^ of GAM, compared with 20 and 30 g L^−1^ of GAM. In addition, the number of ASVs in the culture solution was highest at 10 g L^−1^ of GAM, compared with 20 and 30 g L^−1^ of GAM. These results indicate that a lower medium concentration is recommended as the substrate of the in vitro human oral microbiota simulation model.

**TABLE 1:** The study investigated the microbial composition and diversity in the inoculum (dental plaque and saliva) and at different concentrations of the growth medium (GAM).

	*Porphyromonas*		*Fusobacterium*		ASV		Shannon index	
	(%)		(%)					
	HA	Solution	HA	Solution	HA	Solution	HA	Solution
In vitro model								
GAM 10 g L^−1^	1.21	0.98	3.54	2.23	155	191	6.69	6.97
GAM 20 g L^−1^	0.24	0.00	2.20	1.97	153	166	6.63	6.53
GAM 30 g L^−1^	0.53	0.58	1.71	0.00	168	154	6.57	6.41
Used inoculum	Plaque	Saliva	Plaque	Saliva	Plaque	Saliva	Plaque	Saliva
	1.82 ± 0.60	9.01 ± 0.16	1.45 ± 0.76	5.10 ± 0.91	163 ± 38	176 ± 12	6.83 ± 0.48	6.59 ± 0.08

*Note*: The bacterial genera *Porphyromonas* and *Fusobacterium* are commonly associated with periodontal disease. The bacterial groups were defined using amplicon sequence variants (ASVs).

Abbreviations: GAM, Gifu anaerobic medium; HA, hydroxyapatite.

### Development of BPOM

**FIGURE 2:**
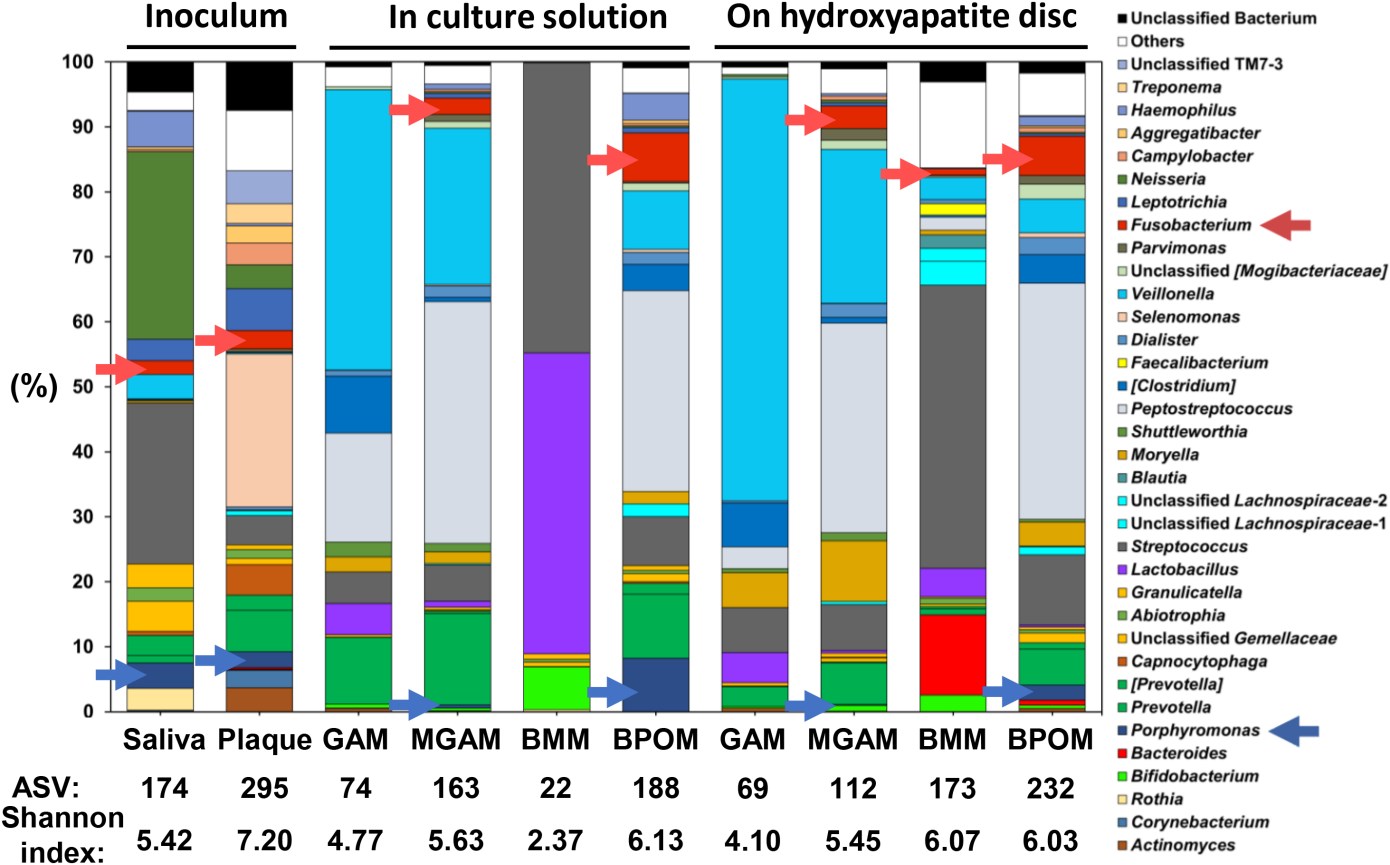
16S rRNA sequencing was employed to investigate bacterial shifts at the genus level in saliva and dental plaque (origin: healthy volunteer) cultured in liquid media and biofilms grown on hydroxyapatite discs. The research utilized four media types: Gifu anaerobic medium (GAM), modified GAM (MGAM), Basal medium mucin (BMM)‐based media and Bio Palette oral medium (BPOM). Genera with lower abundance (<1.0%) and lower similarity (<97%) were included as ‘Others’ and ‘Unclassified Bacteria,’ respectively. *Porphyromonas* and *Fusobacterium* were identified using blue and red arrows, respectively. The amplicon sequence variant number and Shannon index of each sample are also reported. These findings shed light on microbial diversity and community structure in different media types and provided insights for further research.

The growth of bacteria related to the *Porphyromonas* genus was lower in the GAM‐based medium when compared with human saliva and dental plaque (Table 1). To overcome this issue, we developed a new medium, namely BPOM, which contained low nutrient concentrations (0.02% w v^−1^ carbon and <1% nitrogen sources) (Table S1, Supporting Information). BPOM composition referred to the Supplemented Tryptic Soy Broth (American Type Culture Collection Medium 2722). BPOM included hemin and vitamin K1, which are essential for supporting the growth of strict anaerobes (Gibbons & Macdonald, 1960), as well as divalent cations such as calcium and magnesium that facilitate ionic bridging between bacterial cells through interactions with negatively charged cell membranes and other biopolymers in the matrix (Kerchove & Elimelech, 2008). We evaluated BPOM compared with GAM, MGAM and BMM‐based media, the last previously used as an analogue of saliva (Montelongo‐Jauregui et al., 2016). After 72 h of cultivation, the microbiota compositions in the BPOM, GAM, MGAM and BMM‐based media, as well as the inoculum (saliva and dental plaque), were analysed. The genus‐level distribution of sequences based on 16S rRNA genes is shown in Figure 2.

*Porphyromonas* and *Fusobacterium* were detected in original saliva (3.9% and 2.2%, respectively) and dental plaque (2.4% and 2.8%, respectively). Notably, the proportion of bacteria related to *Porphyromonas* was found to be highest in the culture solution and on HA disc with BPOM (8.2% and 2.3%, respectively), as compared with GAM, MGAM and BMM‐based media. Additionally, the proportion of *Fusobacterium* was highest in the culture solution and on HA discs with BPOM (7.5% and 6.0%, respectively) compared with GAM, MGAM and BMM‐based media. However, *Rothia* and *Neisseria* in original saliva exhibited a significant reduction in all types of culture solutions. ASV number and Shannon index in culture solution with BPOM and MGAM‐based medium were high and comparable to those in the original saliva. Moreover, the ASV number and Shannon index in BPOM and BMM on the HA disc were similar to those in the original dental plaque. Thus, we selected BPOM with a high species number and microbial diversity in the culture solution and biofilm on HA for subsequent experiments.

### Preparation of a glycerol mixture from multiple subjects for long‐term storage of *Porphyromonas* and *Fusobacterium* in an in vitro oral microbiota model

To ensure reproducible results, glycerol stocks were prepared from an in vitro oral microbiota model for long‐term storage. Initially, glycerol stocks were prepared from the culture solution inoculated with saliva and dental plaque from one patient and three volunteers in the BPOM. These stocks were further cultured, and glycerol stocks were again prepared from each culture solution. Then, the first four glycerol stocks were mixed, cultured and used to prepare glycerol stock to ensure generalizability. This stock was then cultured again, and glycerol stock was prepared for further use. A diagram showing the process is provided in Figure S1 (Supporting Information).

**FIGURE 3:**
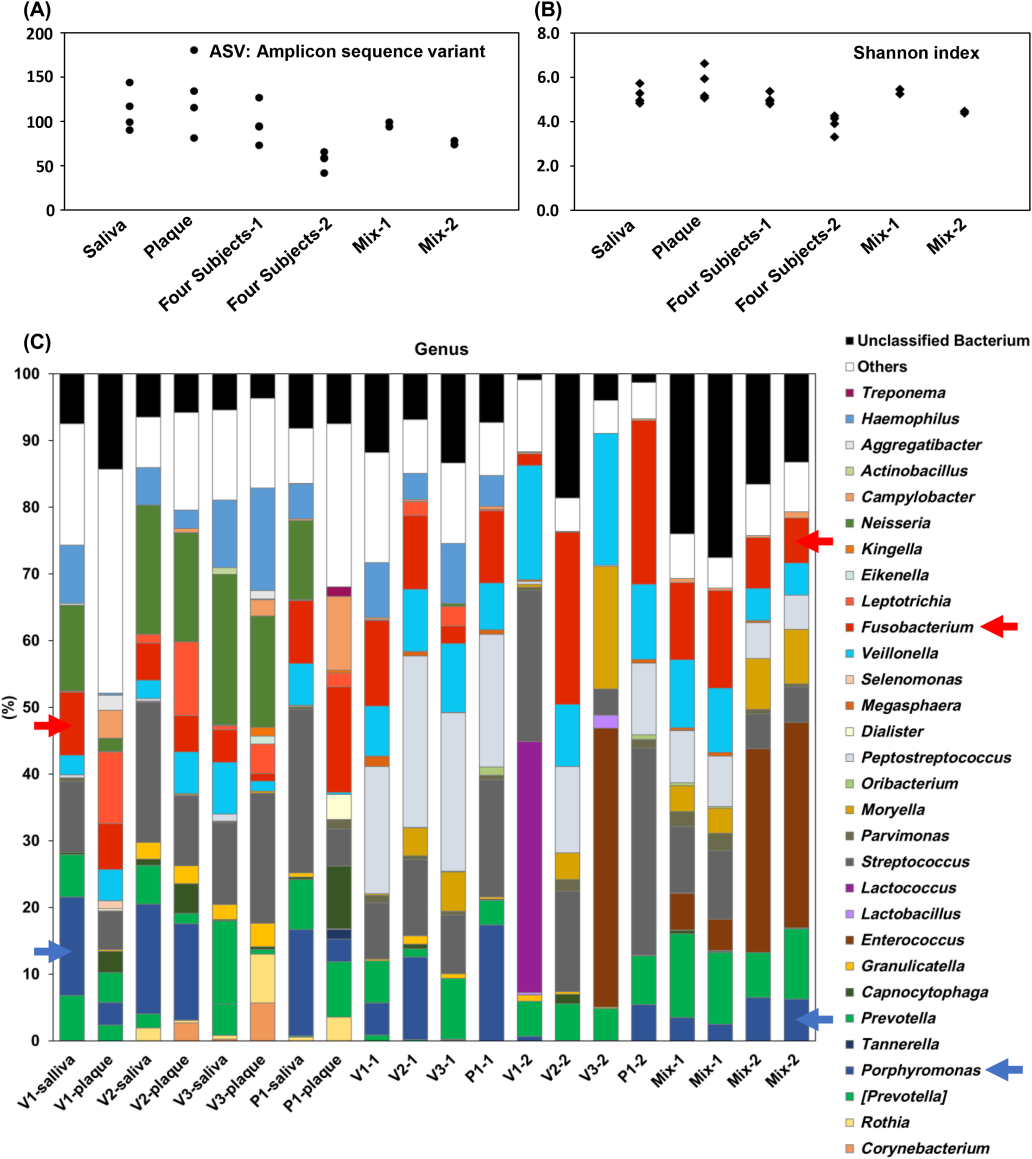
(A) The amplicon sequence variant (ASV) number, representing the total number of unique variants detected in the samples, was determined. (B) The Shannon index, which measures the diversity of bacterial communities, was calculated. Higher values indicate greater diversity. (C) The genus‐level distributions of sequences based on 16S rRNA genes were analysed in inoculums derived from saliva and dental plaque (V1, V2, V3 and P1), as well as in corresponding culture solutions (V1, V2, V3, P1 and Mix 1 and 2). Genera with an abundance below 1.0% and similarity below 97% were classified as ‘Others’ and ‘Unclassified Bacteria,’ respectively. *Porphyromonas* and *Fusobacterium* highlighted using blue and red arrows, respectively, were particularly interesting due to their known roles in oral health and disease.

Microbial community compositions in inoculums and corresponding culture solutions of in vitro human oral microbiota simulation models were analysed based on 16S rRNA gene sequencing. The ASV numbers and Shannon indices were found to be reduced in glycerol stocks stored at −80°C, as evidenced by a comparison of four subjects‐1 and four subjects‐2 or Mix‐1 and Mix‐2 (Figure 3A,B). However, the ASV number and Shannon index of Mix‐2 were higher than those of four subjects‐2. Hence, it can be inferred that mixing four glycerol stocks from different subjects prevented a significant decrease in the ASV number and Shannon index of the culture solution. The genus‐level distributions of bacteria are shown in Figure 3C.

In the inoculum, *Porphyromonas* and *Fusobacterium* accounted for 0.0%–16.5% and 1.1%–15.8% of the community, respectively. The abundance of *Porphyromonas* decreased in all subjects after glycerol stock and near 0% for V1‐2, V2‐2 and V3‐2. *Fusobacterium* was significantly reduced to 1.7% in V1‐2 and was undetected in V3‐2. However, the abundance of *Porphyromonas* and *Fusobacterium* was maintained at more than 6.2% and 6.7%, respectively, in Mix‐2.

### Assessing the effect of minocycline hydrochloride and sitafloxacin on *P. gingivalis* : An in vitro oral microbiota simulation model

Minocycline, a tetracycline derivative, is known for its broad‐spectrum inhibitory activity against bacteria (Morozumi et al., 2022). Sitafloxacin, a fluoroquinolone, is also recognized for its potent antimicrobial activity against various microorganisms (Nakajima et al., 2016). Here, we aimed to evaluate the effects of minocycline hydrochloride and sitafloxacin on *P. gingivalis*. The antibiotics were administrated at 0.1 and 1.0 μg mL^−1^ in our in vitro oral microbiota simulation model, which was inoculated with glycerol stock, Mix‐1. A model without antibiotic administration was used as a control. The administrated concentrations were based on plasma concentrations after oral administrations (0.11 μg mL^−1^ at 2 h after 100 mg dose for minocycline hydrochloride and 0.91 μg mL^−1^ at 3–4 h after 200 mg dose for sitafloxacin) (Jain et al., 2009; Paiboonvong et al., 2018). We added exogenous 10^7^ cells mL^−1^ of *P. gingivalis* to the model at 0 h of cultivation because *P. gingivalis* was not detected in Mix‐2 based on species‐level analysis. After 72 h of cultivation, we quantified the amounts of *P. gingivalis* on HA discs using real‐time PCR analysis.

**FIGURE 4:**
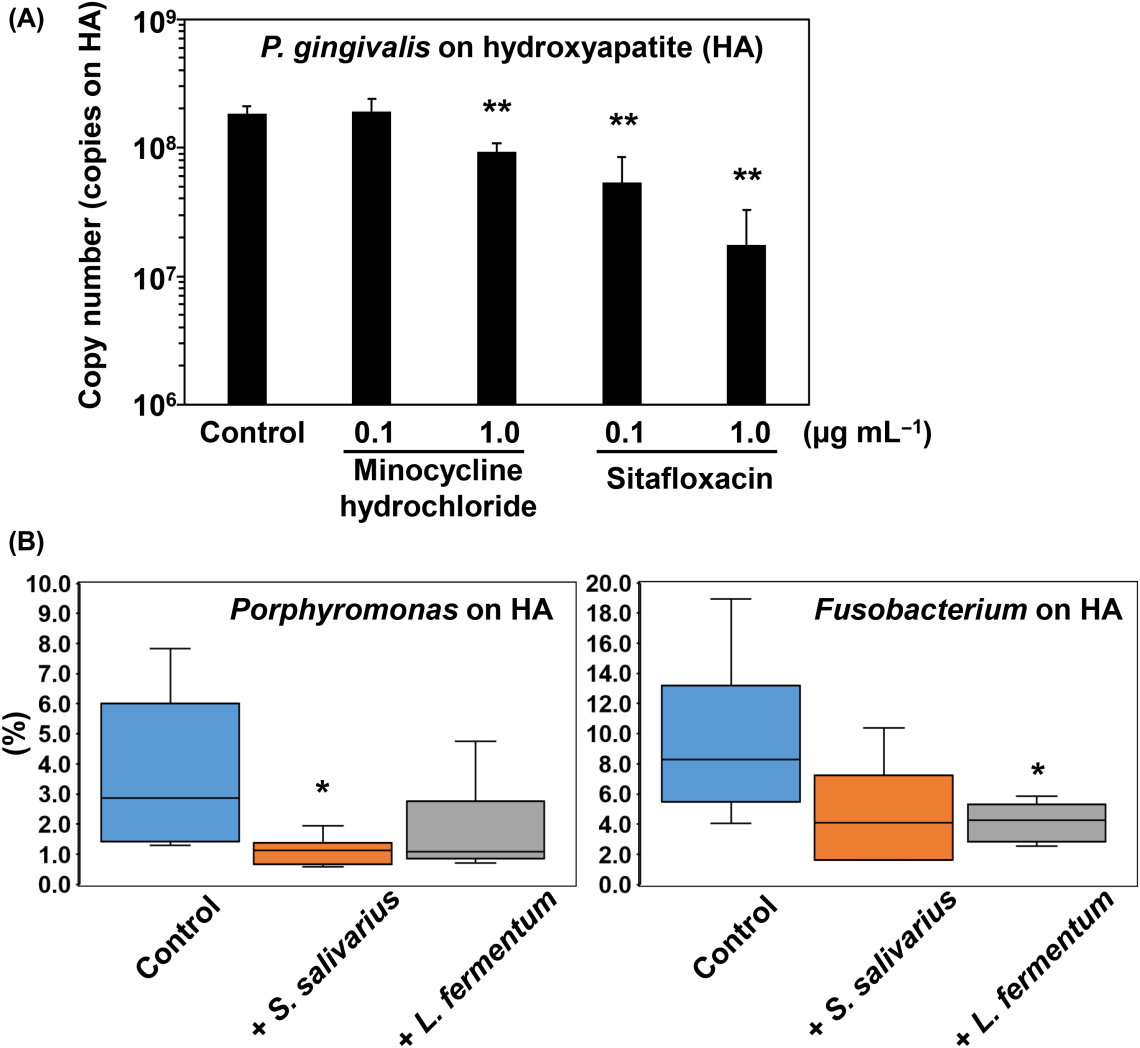
(A) The amounts of *Porphyromonas gingivalis* on hydroxyapatite (HA) discs were measured using Mix‐1: Control (no antibiotics administered), minocycline hydrochloride at 0.1 and 1.0 μg mL^−1^ and sitafloxacin at 0.1 and 1.0 μg mL^−1^. Statistical differences between each group and the Control were calculated using Student's *t* test, and significance was defined as *p* < 0.01 (denoted by double asterisks). (B) Box‐and‐whisker plots were used to display the effects of isolated *Streptococcus salivarius* and *Limosilactobacillus fermentum* administration (or no administration, Control) on the levels of *Porphyromonas* and *Fusobacterium*. The inoculums were collected from three patients and three volunteers. Paired *t*‐tests were conducted to determine statistical differences between each group and the Control, and significance was defined as *p* < 0.05 (denoted by single asterisk).

Our findings suggested that both minocycline hydrochloride and sitafloxacin at the tested concentrations significantly inhibit the growth of *P. gingivalis* on the HA in a dose‐dependent manner (Figure 4A). Although further studies are needed to determine the clinical implications of these results, in the in vitro oral microbiota simulation model, sitafloxacin showed more potent inhibitory effects on *P. gingivalis* than minocycline hydrochloride.

### Assessing the effects of probiotics on *Porphyromonas* and *Fusobacterium* : An in vitro oral microbiota simulation model

Strains of *S. salivarius* and *L. fermentum* were isolated from human saliva and faeces, respectively, and bacteria related to these strains are recognized as probiotics (Ferreira et al., 2022; Wescombe et al., 2012). We evaluated the effects of these probiotics, administrated at a concentration of 4 × 10^7^ cells mL^−1^, on *Porphyromonas* and *Fusobacterium* using our in vitro human oral microbiota simulation model. Inocula were obtained from three patients and three volunteers, each consisting of saliva and dental plaque samples. Administration of *S. salivarius* and *L. fermentum* did not affect the amount of biofilm on the HA disc (Figure S2: Supporting Information). Administration of *S. salivarius* significantly reduced the proportion of *Porphyromonas* in the biofilm on the HA disc (*p* = 0.038 by paired *t*‐test). In contrast, administration of *L. fermentum* led to a significant decrease in the proportion of *Fusobacterium* (*p* = 0.045 by paired *t*‐test) on HA discs (Figure 4B). Fixation of these probiotics was ascertained by an increase in genera *Streptococcus* or *Lactobacillus*.

## DISCUSSION

Our present study aimed to develop an in vitro oral microbiota simulation model, which involved the development of a synthetic medium called BPOM, with relatively low nutrients, that is, 0.02% w v^−1^ carbon and <1% nitrogen sources. A modified SHI medium, previously developed by Lamont et al. (2021) incorporated 0.1% sucrose and 10% fetal bovine serum and was rich in nutrients but had been shown to decrease phyla *Bacteroidota* and *Fusobacteria*, including *Porphyromonas* and *Fusobacterium*, respectively. Furthermore, a study using the Calgary Biofilm Device with a synthetic medium containing a higher concentration of serum (20%) observed a decrease in species diversity when inoculated with human dental plaque (Baraniya et al., 2020). Our result showed that a lower medium concentration and lower glucose content (0.02% w v^−1^) as a carbon source was favourable for the growth of target bacteria, *Porphyromonas* and *Fusobacterium*, and for promoting microbiota diversity in culture solution and oral biofilm on HA discs in our batch culture. These findings agreed with the previous result using continuous culture, showing that more *F. nucleatum* was present and a more diverse community was obtained in a glucose‐limited chemostat (1% w v^−1^ glucose), compared with glucose‐excess chemostat (3.5% w v^−1^ glucose) (Marsh et al., 1983). The low glucose content in BPOM or low concentrations of GAM would be indirectly advantageous for *Porphyromonas* and *Fusobacterium*, which are generally asaccharolytic or weakly fermentative, according to previous studies (How et al., 2016; Robrish et al., 1991). However, it should be noted that supragingival sites also contain saccharolytic microorganisms, among other genera (Takahashi, 2005).

We evaluated the utility of glycerol stock prepared from BPOM liquid culture inoculated with human saliva and dental plaque. Typically, glycerol stocks are directly prepared from saliva collected from a human donor (Li et al., 2021). Some bacterial species were lost during the cryopreservation of the artificial oral microbiota, decreasing bacterial diversity. Mixing multiple glycerol stocks prepared from inoculums collected from different human subjects was also found to reduce the decline in bacterial species and diversity during cryopreservation but maintain high ratios of target bacteria, including *Fusobacterium* and *Porphyromonas*. This glycerol stock mixture could serve as an alternative to human saliva and dental plaque, provide a large volume and allow for experiments with improved reproducibility.

Next, we compared the antimicrobial activity of sitafloxacin and minocycline hydrochloride. Sitafloxacin, an antibiotic used in Japan and Thailand, is commonly prescribed for respiratory and urinary tract infections (Chen et al., 2020). This medication has recently become available in China as well (Wu et al., 2021). During supportive periodontal therapy, it has proven to be as effective as azithromycin, a traditional antibiotic. This is particularly beneficial for patients for whom invasive mechanical treatment is not suitable (Nakajima et al., 2016). In comparison to minocycline hydrochloride, sitafloxacin demonstrated a significantly lower minimum inhibitory concentration against the majority of tested Gram‐negative bacterial strains (Zhou et al., 2022). Moreover, sitafloxacin displayed higher bactericidal activity than minocycline hydrochloride against the Gram‐negative strain *P. gingivalis* (Tomita et al., 2014).

This study evaluated the efficacy of probiotics, *S. salivarius* and *L. fermentum*, in disrupting biofilm containing complex microbiota. *S. salivarius* has been shown to exhibit antibacterial activity against a range of oral bacteria, including *P. gingivalis*, *P. endodontalis* and *F. nucleatum* (Moon et al., 2016), and can co‐aggregate with both *P. gingivalis* and *F. nucleatum* (Lévesque et al., 2003). Although *F. nucleatum* is known to interact with a greater variety of oral microbes than *P. gingivalis* in dental plaque biofilms (Lasserre et al., 2018), the results of this study suggest that *S. salivarius* is particularly effective at inhibiting the interaction of *Porphyromonas* in the biofilm. In addition, *L. fermentum* was found to reduce the abundance of *Fusobacterium* more than *Porphyromonas* in the biofilm, likely due to the production of acid substances by *L. fermentum*, which creates an unfavourable acidic environment that more severely impedes the early colonization of *F. nucleatum* compared to *P. gingivalis* (Zhang et al., 2021; Zilm et al., 2002).

However, the model has a limitation in that it is operated under anaerobic conditions, leading to the loss of certain aerobic/facultative anaerobic bacteria such as *Rothia* and *Neisseria* (Monnoyer et al., 2021). In addition, in vitro models have limitations due to their lack of actual anatomy. Therefore, combining in vivo studies would be necessary to understand the effects on intact physiological processes in preclinical phases (Xu et al., 2021).

In conclusion, the oral microbiota simulation model we have developed may serve as an efficient and reproducible in vitro screening platform for evaluating the efficacy of antimicrobial drugs, probiotics and live biotherapeutic products.

## AUTHOR CONTRIBUTIONS

**Kengo Sasaki:** Conceptualization (lead); data curation (lead); methodology (equal); supervision (lead); visualization (equal); writing – original draft (lead). **Yasunobu Takeshima:** Conceptualization (lead); investigation (equal); methodology (equal); supervision (equal); writing – original draft (equal). **Ayami Fujino:** Data curation (equal); software (lead). **Junya Yamashita:** Resources (lead); validation (equal). **Akira Kimoto:** Resources (equal); validation (lead); writing – review and editing (equal). **Daisuke Sasaki:** Formal analysis (lead); software (equal). **Akihiko Kondo:** Formal analysis (equal); funding acquisition (lead); project administration (equal). **Masaya Akashi:** Project administration (equal); resources (equal); visualization (lead). **Ryo Okumura:** Conceptualization (equal); investigation (equal); project administration (lead); writing – review and editing (lead).

## CONFLICT OF INTEREST STATEMENT

Kengo Sasaki, Yasunobu Takeshima, Ayami Fujino and Ryo Okumura are employees of Bio Palette Co., Ltd. All other authors declare no competing interests.

## ETHICS STATEMENT

All subjects provided written informed consent before specimen collection. All methods used in this study were performed by the Declaration of Helsinki.

## Supporting information


**Table S1.** Used medium composition (L^−1^). Bio Palette oral medium: BPOM, Gifu anaerobic medium: GAM, modified GAM: MGAM and Basal medium mucin: BMM.
**Figure S1.** Flow chart of glycerol stock preparations. Duplicate glycerol stock (Mix1‐1 and Mix2‐1) was prepared from the culture solution inoculated with a glycerol stock mixture of V1, V2, V3 and P1. Microbiota compositions were analysed for red samples.
**Figure S2.** Biofilm mass on the HA disc determined by crystal violet binding assay. The optical density (OD) at 595 nm was measured. Statistical differences between each group and Control were not observed using the paired *t*‐test (*p* > 0.05).

## Data Availability

The raw sequencing data generated in this study were deposited into the DDBJ, EMBL and Genbank databases (http://getentry.ddbj.nig.ac.jp) with the accession numbers DRR420819–DRR420878 under the BioProject PRJDB14784.
