# Supplementary material for: Construction of a versatile in vitro cultivation screening platform using human oral microbiota
Source: Environ Microbiol Rep. 2024 Feb 29;16(2):e13243. doi: 10.1111/1758-2229.13243 (PMC10904971; doi:10.1111/1758-2229.13243)
Supplement: Supplementary file 1 — Table S1. Used medium composition (L−1). Bio Palette oral medium: BPOM, Gifu anaerobic medium: GAM, modified GAM: MGAM and Basal medium mucin: BMM. Figure S1. Flow chart of glycerol stock preparations. Duplicate glycerol stock (Mix1‐1 and Mix2‐1) was prepared from the culture solution inoculated with a glycerol stock mixture of V1, V2, V3 and P1. Microbiota compositions were analysed for red samples. Figure S2. Biofilm mass on the HA disc determined by crystal violet binding assay. The optical density (OD) at 595 nm was measured. Statistical differences between each group and Control were not observed using the paired t‐test (p > 0.05). [file EMI4-16-e13243-s001.docx]

# Supporting Information

**Table S1.** Used medium composition (L^–1^). Bio Palette oral medium: BPOM, Gifu anaerobic medium: GAM, modified GAM: MGAM, and Basal medium mucin: BMM.

| Ingredients | BPOM | GAM-based | | MGAM-  based | | BMM-based |  |
| --- | --- | --- | --- | --- | --- | --- | --- |
| Tryptone | 5.67 g |  | |  | | 2.5 g |  |
| Peptone |  | 1.69 g | | 1.20 g | |  |  |
| Protease peptone |  | 1.69 g | | 1.20 g | | 5.0 g |  |
| Digested serum |  | 2.29 g | | 2.40 g | |  |  |
| Mucin |  |  | |  | | 6.25 g |  |
| Stone | 1.00 g | 0.51 g | | 0.72 g | |  |  |
| Liver extract |  | 0.20 g | | 0.29 g | |  |  |
| Beef extract |  | 0.37 g | | 0.53 g | |  |  |
| Yeast extract | 1.67 g | 0.85 g | | 0.60 g | | 2.5 g |  |
| Glucose | 0.20 g | 0.51 g | | 0.12 g | | 0.20 g |  |
| Soluble starch |  | 0.85 g | | 1.20 g | |  |  |
| NaCl | 1.67 g | 0.844 g | | 0.72 g | | 0.844 g |  |
| KCl | 1.20 g | 1.20 g | | 1.20 g | | 1.25 g |  |
| CaCl2·2H2O | 0.146 g | 0.146 g | | 0.146 g | | 0.146 g |  |
| MgCl2 | 0.052 g | 0.052 g | | 0.052 g | | 0.052 g |  |
| K2HPO4 | 0.83 g | 0.42 g | | 0.60 g | | 0.342 g |  |
| L-Cysteine·HCl | 0.50 g | 0.05 g | | 0.07 g | |  |  |
| L-Cysteine |  |  | |  | | 0.25 g |  |
| L-Arginine |  |  | | 0.24 g | |  |  |
| L-Tryptophan |  |  | | 0.05 g | |  |  |
| Hemin | 5.0 mg |  | | 2.4 mg | | 5.0 mg |  |
| Vitamin K1 | | 1.0 mg | |  | 1.2 mg | 1.0 mg | |
| Sodium thioglycollate | |  | | 0.05 g | 0.072 g |  | |
| Antifoam | | 50 µL | | 50 µL | 50 µL | 50 µL | |
| 1% Resazurin | | 0.1 mL | | 0.1 mL | 0.1 mL | 0.1 mL | |


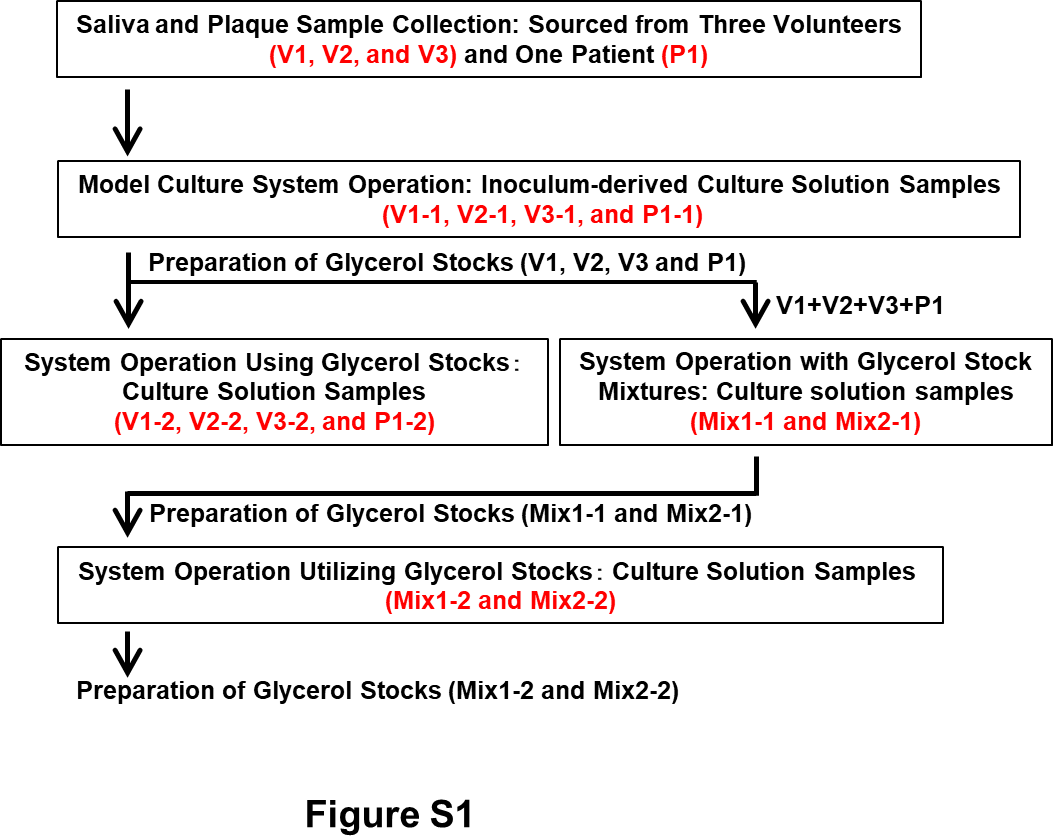


**Figure S1.** Flow chart of glycerol stock preparations. Duplicate glycerol stock (Mix1-1 and Mix2-1) was prepared from the culture solution inoculated with a glycerol stock mixture of V1, V2, V3, and P1. Microbiota compositions were analyzed for red samples.


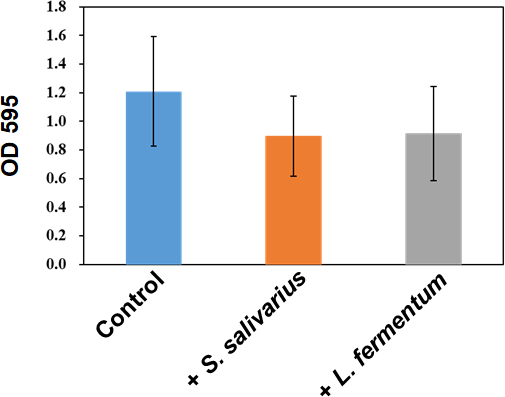


**Figure S2.** Biofilm mass on the HA disc determined by crystal violet binding assay. The optical density (OD) at 595 nm was measured. Statistical differences between each group and Control were not observed using the paired t-test (p > 0.05).
